# Does personality matter? Exploring its moderating role on the relationship between neighbourhood ethnic outgroup-size and preferences for Brexit

**DOI:** 10.1080/14616696.2023.2277279

**Published:** 2023-11-06

**Authors:** Franco Bonomi Bezzo, Laura Silva, James Laurence, Katharina Schmid

**Affiliations:** a Department of Social and Political Science, University of Milan, Milan, Italy; b Institut national d'études démographiques (INED), Paris, France; c Labour and Public Economics Unit, Paris School of Economics, Paris, France; d Sciences Po, CRIS, CNRS, Paris, France; e Economic and Social Research Institute, Dublin, Ireland; f UCL Social Research Institute, University College London, London, UK; g Department of People Management and Organisation, Universitat Ramon Llull, Esade Business School, Barcelona, Spain

**Keywords:** Neighbourhood, ethnic outgroup-size, Brexit, personality, political behaviour

## Abstract

Prior research has examined the relationship between ethnic outgroup-size at the neighbourhood level and Brexit support, yet there is a lack of understanding on the factors that moderate these effects. This paper critically extends prior debate by focusing on how personality traits moderate not only the extent to which the levels (2011) of ethnic outgroup-size in individuals’ residential neighbourhoods but also the increase thereof (2001-2011) are associated with individuals’ preferences about the 2016 Brexit referendum. Using data from Understanding Society, we find that two personality traits, agreeableness and openness, are key moderators affecting the above-mentioned relationship. High-agreeable and high-open individuals are less likely than low-agreeable and low-open individuals to support Brexit. However, while the gap between low and highly agreeable individuals shrinks as ethnic outgroup-size increases, the gap widens between those higher vs. lower in openness. Our findings highlight the multifaceted role of personality traits as a driver of heterogeneous effects on political behaviour. In sum, this paper shows that analysing the complex and intertwined nature of both contextual and individual factors is fundamental for a better understanding, not only of the Brexit referendum but, more broadly, of anti-immigrant sentiment.

## Introduction

Immigration into the UK, from an increasingly diverse range of countries, has kept rising markedly since the beginning of the 2000s (The Migration Observatory, 2016), leading cities and neighbourhoods to become progressively more ethnically diverse. Social science scholars have thus widely investigated the conditions determining the attitudes of host communities towards this rise in ethnic outgroup-size. There is mixed evidence about how community outgroup-size^1^ affects intergroup attitudes (Kaufmann and Harris 2015; Bond and Tejeiro 2019; Kawalerowicz 2021) and trust (Putnam 2007), with findings showing either improvement, deterioration, or non-effects (Schmid *et al*. 2014; Kros and Hewstone 2020; Schlueter and Scheepers 2010; Bowyer 2008; Lubbers and Scheepers 2007). One possible explanation for such mixed evidence is that outgroup-size exerts heterogeneous effects on different individuals. Prior work, for example, has shown that outgroup-size is associated with more negative attitudes, especially for people from lower socio-economic backgrounds, living in social housing, or for individuals who have no intergroup contact with ethnic minority members (Meleady *et al*. 2017). Extending this prior work, the present paper examines another possible driver of the heterogeneous effects of outgroup-size: individual differences in personality traits.

The contribution of this paper is threefold. First, we examine how outgroup-size across communities affects residents’ anti-immigrant sentiment from a *policy-related behavioural* perspective rather than just an attitudinal one (Silva *et al*. 2023). Although attitudes and behaviour are interconnected, they remain distinct constructs within the domains of psychology and social sciences (LaPiere 1934; Wicker 1969). Critically, individuals’ attitudes (e.g. individuals’ perceptions or feelings about immigration) do not always predict how they act (e.g. voting behaviour in an election or the like), often reflecting an inconsistency between attitudes and behaviour (Liska 1974). In other words, simply because an individual holds a particular attitude about a political issue does not mean that they will behave in a way that reflects this attitudes (Mughan and Paxton 2006). Therefore, to fully understand how the rise in ethnic outgroup-size shapes societies, studies need to not only explore attitudinal perceptions about outgroup-size (the focus of much of prior research in this area, e.g. Silva *et al*. 2023), but critically, also needs to consider behavioural responses to it. To do so, we focus on the 2016 Brexit referendum, a key political event with a wide range of economic, social and political consequences for the UK. Research has shown that Brexit support was driven by multiple factors, of which anti-immigrant sentiment was just one – albeit a key – driver (Clark *et al*. 2017; Goodwin and Milazzo 2017; Colantone and Stanig 2018; Green *et al*. 2022). However, we contend that the primary reason why community outgroup-size *in particular* was linked to support for/against leaving the EU was due to its association with people’s anti-immigrant sentiments. In other words, support for Brexit acts as an effective proxy for anti-immigrant sentiment when it comes to analysing the effects of outgroup-size, and we provide empirical evidence in support of this in the paper.

Second, we test the moderating role of individual differences in *personality*, based on the Big 5 Model of Personality (McCrae and Costa 1985): extraversion, agreeableness, conscientiousness, emotional stability and openness to experience. Third, we investigate how both *levels* and the *increase* in outgroup-size over time have shaped such political behaviours, and the moderating impact of personality traits on these relationships.

We posit that individual differences in personality may condition reactions to outgroup-size in the immediate residential environment. This idea is consistent with established theories in the realm of political psychology, according to which individuals respond differently to various situational triggers (Feldman 2003; Dinesen *et al*. 2016). Previous studies have shown, for example, that traits such as extraversion or agreeableness may shape individuals’ perceptions and views about ethnic differences (Sibley and Duckitt 2008). Extending this work further, we argue that some individuals may react more negatively than others to increasing outgroup-size – depending on their unique personality traits.

Our analysis highlights that personality traits play an important, yet complex, role in moderating the relationship between neighbourhood outgroup-size and a key policy manifestation of individuals’ anti-immigrant sentiment, captured here by people’s preferences for remaining or leaving the EU in the Brexit referendum. Agreeableness and openness, in particular, are the personality traits that seem to have the strongest impact in moderating this relationship.

The remainder of the article continues as follows. The next section outlines the theoretical background for our analysis. The following section elaborates on our data and methods. After this we first present our results, before we discuss our findings and draw some general conclusions.

## Theoretical background

The extent to which individuals hold positive or negative views on outgroup-size depends on a variety of factors, including characteristics at both the contextual and the individual level (Abreu and Öner 2020). The literature investigating the effect of contextual variables, such as outgroup-size, on the one hand, and of individual characteristics such as personality, on the other hand, on immigration-related preferences is vast (Johnston *et al*. 2015; Danckert, *et al*. 2017; Ziller and Hewstone 2019; Dinesen *et al*. 2016; Ackermann *et al*. 2018). However, research at the nexus of these three dimensions is more limited, even though the three research areas are tightly inter-connected. Some studies have focused on the interaction between outgroup-size at the local level and attitudes towards immigrants for individuals characterised by an authoritarian personality, who, according to Adorno *et al*. (1950), are typically highly conservative and particularly sensitive to change. Van Assche *et al*. (2014) for example find that higher neighbourhood diversity predicts less positive attitudes toward immigrants among individuals scoring higher in authoritarianism. Similarly, Velez and Lavine (2017) find that higher levels of contextual ethnic diversity are related to lower negative immigration beliefs and political intolerance for those scoring low in authoritarianism, but not for those scoring high on the same trait. Focusing on the Big 5 personality traits, a study by Danckert *et al*. (2017) using survey data from Denmark and Canada, found that openness positively moderates the effect of interethnic encounters on immigration attitudes. However, this study considered individual-level, self-reported measures of intergroup encounters rather than contextual diversity data. Research from the United States (Johnston *et al*. 2015) shows that personality traits that relate to risk aversion moderate the effect of local ethnic change on the perceived cultural threat from immigrants and on immigration-related policy preferences. Recently, Silva *et al*. (2023) investigated the extent to which personality traits moderate the relationship between both absolute levels and changes in ethnic outgroup-size at the local level, respectively, and prejudicial attitudes, measured by capturing people’s level of comfort with having ethnic outgroups in various domains of their social environments, e.g. in their neighbourhood or child’s school. This analysis was based on a sample of middle-aged adults in the UK. However, to our knowledge, little research has so far considered the impact of individual personality traits on the relationship between both levels of, and changes in, outgroup-size at the contextual-level on political intended behaviours. While measuring prejudicial attitudes is valuable and informative, Silva *et al*. (2023) fail to capture actual intended behaviours. Simply holding an attitude does not guarantee that it will manifest as an associated behaviour (Wallace *et al*. 2005), and psychological studies have shown that intentions are a stronger predictor of behaviour than mere attitudes (Armitage and Christian 2003). The behavioural measure we use in the current paper offers therefore greater validity over classic self-reported measures, as it taps a real-life outcome with actual consequences for policy and society.

Attitudes and behaviours may exhibit a mutual influence on each other, whereby an individual's attitude can shape their behaviour, yet conversely, engaging in specific behaviours can also impact a person's attitudes or beliefs regarding that conduct (Schuman and Johnson 1976; Azen *et al*. 2018). However, as argued above, attitudes and behaviours are often not perfectly aligned. Attitudes reflect a person's evaluations, thoughts and sentiments towards an issue, person or object, while behaviours encompass actions and demeanour. Attitudes encompass a person's comprehensive evaluation or judgment of a particular object, person, group, idea, or event, typically reflecting an internal mental construct. Conversely, behaviours constitute evident actions or conduct that can be observed, measured, and analysed. Furthermore, both attitudes and behaviours are subject to moulding and modification through diverse factors like personal experiences, social interactions, education, and persuasive communication. Attitudes can demonstrate relative stability over time; however, they can undergo transformation due to novel information, life experiences, or persuasive communication. On the other hand, behaviours may vary contingent on situational factors, context, societal norms, and personal motivations, occasionally diverging from attitudes due to external influences or conflicting elements (Schuman and Johnson 1976). Given these differences between attitudes and behaviour, solely analysing attitudinal responses to demographic change does not tell us everything we need to know about how demographic changes may influence people’s behaviours.

Understanding the link between personality traits and support for Brexit also provides valuable insights into the psychological factors at play during critical political events. It provides insights into whether individual personality traits may influence not only individuals’ self-declared attitudes but also how people perceive and respond to significant political decisions, highlighting the multidimensional nature of political behaviours and the need for a comprehensive understanding of the underlying psychological mechanisms. Further research in this area can deepen our understanding of the role of personality in shaping political ideologies and decisions.

## Support for Brexit and anti-immigrant sentiment

Research has shown that support for the UK to leave the EU had multiple drivers, including political marginalisation, negative attitudes towards the EU, and economic deprivation and decline (Clark *et al*. 2017; Colantone and Stanig 2018; Green *et al*. 2022). One key driver that has emerged from the literature, however, is anti-immigrant sentiment and national identity, which, alongside authoritarian values, played a key role in Brexit support (Curtice 2017; Goodwin and Milazzo 2017). In addition, some research suggests that Brexit support may have been influenced in particular by threats concerning immigration from Eastern European countries into the UK (Martin *et al*. 2019). Throughout the years prior to the referendum, immigration-related concerns increased due to a rising net immigration into the UK, in part, driven by the EU enlargement in 2004 which provided free access to the European borders to eight Eastern European countries (Watt and Wintour 2015; Dustmann *et al*. 2003). Previous studies have confirmed that both the perception of a general threat and a negative view of immigration predicted voting behaviour in the referendum (Abrams and Travaglino 2018; Golec de Zavala *et al*. 2017). Respondents who expressed concerns about immigration into the UK were more likely to vote Leave (Hobolt 2016; Goodwin and Milazzo 2017), and in a national poll immediately following the referendum, 33% of Leave voters cited ‘regaining control over immigration’ as their main motivation (Ashcroft and Bevir 2016).

While support for Brexit was driven by a variety of other factors (Clarke *et al*. 2017; Colantone and Stanig 2018; Green *et al*. 2022), we argue that outgroup-size at the local level shaped support for Brexit through an ‘anti-immigration’ pathway. To further support this idea with empirical data, we undertook an analysis of the 2017 British Election Study data, which contains both a measure of support for leaving the EU (vote in the Brexit referendum) and a measure anti-immigrant sentiment (how strongly individuals feel that ‘too many immigrants are let into this country’), alongside the key predictors used in this study. This analysis shows that nearly 60 per cent of the association between our measures of outgroup-size and support for leaving the EU can be explained by their association with anti-immigrant sentiment (particularly interesting given we only control for one indicator of anti-immigrant sentiment; see Appendix A.1 for full discussion of the approach, analysis, and results). In sum, this provides strong evidence that, when it comes to exploring the effects of ethnic outgroup-size, intended Brexit support represents an effective manifestation of anti-immigrant sentiment with behavioural and policy underpinnings.

## Neighbourhood outgroup-size and policy manifestation towards immigration

Recent studies have started to examine the extent to which the characteristics of local communities, in particular their ethnic outgroup-size, are linked with anti-immigrant sentiment, the EU, and the EU Referendum results. Intergroup contact, i.e. the existence and type of interactions between different ethnic groups within the same community, plays a central role in explaining this relationship. According to contact theory (Allport 1954; Pettigrew 1998; Pettigrew and Tropp 2006), when outgroup-size increases within one’s local area there are greater opportunities for face-to-face interaction and the development of social ties and friendships between different ethnic groups. Engaging with diverse individuals would thus lead to less hostile attitudes towards what is perceived as different (Schlueter and Scheepers 2010), although others have highlighted that ethnic diversity can also increase opportunities for negative intergroup encounters, which can generate greater prejudice and anti-immigrant sentiment (Laurence and Bentley 2018). At the same time, increasing ethnic mixing may also constitute a source of threat to the majority group’s social and economic standing, which leads to a deterioration of attitudes toward the minority group (Oliver and Wong 2003; Blalock 1967; Quillian 1995). Theories of ethnic competition (Levine and Campbell 1972) similarly stress how individuals, and especially those from lower social groups, shift their support towards far-right ideologies with the ambition to reduce actual or perceived competition from immigrants over scarce resources such as jobs, housing and welfare benefits. Moreover, increasing diversity in individuals’ areas of residence may trigger feelings of loss of community, social disintegration, and societal disaffection. Such feelings can, in turn, stimulate more conservative voting patterns as a protest vote (Coffé *et al*. 2007; Van der Brug *et al*. 2000; Lubbers and Scheepers 2007).

Most of the research on the relationship between inter-ethnic encounters at the local level and support for far-right, anti-immigrant, populist movement has found evidence of a positive relationship between greater inter-ethnic encounters and support for far-right and populist parties (Biggs and Knauss 2012; Bowyer 2008; Kaufmann 2017). The work by Enos (2014, 2016, 2017) is particularly relevant in this respect, providing evidence that increased immigration and higher opportunities for interethnic contact do not translate into more positive attitudes towards immigrant groups, but rather into exclusionary attitudes and in a political shift towards conservativism. However, some authors find the opposite (Evans and Ivaldi 2021) and some even found evidence for both a positive and negative relationship depending on the geographical scale of reference (Green *et al*. 2022; Vasilopoulos *et al*. 2021; Gravelle *et al*. 2021). For example, Vasilopoulos *et al*., (2021) find that individuals in neighbourhoods with high levels of immigration are less likely to vote for the far-right but, at the regional level, higher immigration and unemployment correlate instead with greater support for conservative parties.

Borkowska and Laurence (forthcoming) suggest that while higher levels of ethnic outgroup-size in the local area positively correlate with more tolerant attitudes towards immigrants and lower support for Brexit, a larger recent increase in the immigrant population in the very same local area, positively correlates, with increased anti-immigrant attitudes and pro-Brexit voting intentions, confirming previous findings from Goodwin and Heath (2016). In fact, it is commonly assumed that higher levels of outgroup-size, experienced over a longer period, may increase the chances of positive interactions and, therefore, improve intergroup contact (Kaufmann 2017). Conversely, increasing recent changes in immigrants-share may trigger defensive cultural responses in order to tackle a perceived ‘threat’ (Newman and Velez 2014). Empirical findings from Greece confirm this hypothesis. Using the refugee crisis as a natural experiment, the authors find that local residents reported higher hostility towards refugees and greater support for restrictive asylum and immigration policies in those islands that experienced significant refugee inflows than in similar islands unaffected by the crisis (Hangartner *et al*. 2019). In the UK, Kawalerowicz (2021) also finds that anti-immigrant attitudes are more likely to be expressed by natives who live in constituencies where there has been a large change in diversity between 2001 and 2011. These sudden and unexpected experiences of diversity drive negative attitudes towards immigration that, in turn, may have generated stronger support for Brexit. The latter phenomenon seems particularly relevant given the salience of immigration during the period, with the pro-Brexit campaign strongly centred around the ‘taking back control’ rhetoric, which aimed to depict immigration as a threat to national sovereignty and to the economy (Lee *et al*. 2018). In line with this, Borkowska and Laurence (forthcoming) also find that the length of exposure matters. In the shorter term, recent increases in the local share of immigrants (five years prior to the EU referendum) had no effect on immigration-attitudes nor Brexit-support. However, in line with the existing literature (Goodwin and Heath 2016; Kaufmann 2017; Kawalerowicz 2021), medium-term increases in the ethnic out-group (between 2001 and 2011) were positively associated with both anti-immigrant attitudes and Brexit-support. Based on previous evidence about levels of, and changes in, outgroup size, respectively, and Brexit-support, we thus hypothesise that:

Hypothesis 1: The larger the levels of outgroup-size, the lower the support for Brexit.

Hypothesis 2: The larger the increase of outgroup-size, the stronger the support for Brexit.

## The role of personality traits

Individual differences and personality traits can play a key role in explaining many of our attitudes as well as behaviours (Gerber *et al*. 2011). Such traits are the result of both individuals’ predispositions and environmental influences. Thus, individual differences have been considered largely exogenous to political attitudes and behaviours (Mondak 2010), although some authors have cast doubts on this assumption (Verhulst *et al*. 2012).

A large body of work has shown that individual differences concerning preferences for ideologies surrounding social and economic issues are related to anti-immigrant sentiment. Moreover, in the field of political psychology, individual personality predispositions are critical factors shaping political attitudes and behaviours (Gerber *et al*. 2010; Mondak 2010; Mondak *et al*. 2008). Prior research has shown, for example, that individuals scoring higher in authoritarianism (Altemeyer 1988) and social dominance orientation (Pratto *et al*. 1994), tend to report higher levels of prejudice, and, in policy terms, pro-Brexit vote (Golec de Zavala *et al*. 2017)

In this paper, we focus on one of the most influential models of personality, the Big 5 Personality model (McCrae and Costa 1985). This model captures variation in individual differences along five core dimensions which are posited to underlie most of the variation in human personality. The five core traits are classified as extraversion (i.e. the degree to which individuals are more or less sociable, outgoing and assertive), agreeableness (i.e. the degree to which individuals are more or less good-natured, cooperative and trusting), conscientiousness (i.e. the degree to which individuals are more or less responsible, dependable and organized), emotional stability (i.e. the degree to which individuals are more or less calm, self-confident and secure) and openness to experience (i.e. the degree to which individuals are more or less imaginative, curious and creative).

## Personality traits, policy responses to immigration, and political participation

When it comes to personality traits, the literature suggests that two traits in particular, openness and agreeableness, are critical for the development of attitudes towards immigration and policy responses to immigration (Danckert *et al* 2017). Regarding the other traits, there is less evidence suggesting that these traits play a significant role on our outcome of interest. Nonetheless, we review each of them in turn, providing some tentative hypotheses on their moderating role on how local levels and changes in outgroup-size over time shape political behaviours related to immigration.

The literature looking at the relationship between agreeableness and political behaviour reports mixed findings. Agreeableness has been found to be associated with economic liberalism as well as with social conservatism (Gerber *et al*. 2010; Hirsh *et al*. 2010). Authors have found agreeableness to be negatively correlated with Brexit support (Areal 2021; Lee *et al*. 2018) and positively correlated with support for the EU government and enlarging the EU (Schoen 2007). However, individuals characterised by high agreeableness are prone to social conformity as they are attracted by the communal and cooperative components of group identification (Bakker and De Vreese 2016; Gerber *et al*. 2012: 661). Under this perspective, agreeableness has been associated with national identification (Duckitt and Sibley 2014; Sagiv *et al*. 2012), but not EU identification (Carey 2002) nor trust in EU institutions (Bakker and De Vreese 2016). Overall, we expect that:

Hypothesis 3: Agreeableness is negatively correlated with Brexit support.

At the same time, similar to openness, higher levels of agreeableness have also been associated with more positive ethnicity-related attitudes (Gallego and Pardos-Prado 2014; Jackson and Poulsen 2005). Since agreeableness implies a pro-social orientation, as well as kind and caring attitudes towards others, individuals characterised by high agreeableness should be more likely to engage in positive relationships with others, including people belonging to other ethnic groups, and more willing to empathise with them. Agreeable individuals show a higher level of tolerance toward members of other cultures and groups (Bakker and De Vreese 2016). Ackermann *et al*. (2018) find that agreeableness moderated the role of neighbourhood outgroup-size in Switzerland, whereby agreeable individuals preferred their country to be more open, both economically and culturally. Based on this, we predict:

Hypothesis 3a: Higher agreeableness boosts the (negative) relationship between levels of outgroup-size and support for Brexit.

At the same time, Mondak (2010) finds that high levels of agreeableness, as well as low levels of conscientiousness, are associated with lower risk-aversion and a more favourable propensity towards diversity and social change in general. Accordingly,

Hypothesis 3b: Higher agreeableness attenuates the (positive) relationship between increases of outgroup-size and greater support for Brexit.

Moving on to openness, authors have found evidence for a correlation between this trait and support for the EU, identification with Europe and adoption of the Euro currency (Bakker and de Vreese 2016; Schoen and Schumann 2007). High-openness individuals have also been deemed to be less conservative and more aligned to liberal political beliefs (Carney *et al*. 2008). Evidence looking at the Brexit referendum specifically points to a robust negative relationship between openness and probability to support Brexit (Garretsen *et al*. 2018; Lee *et al*. 2018; Shuttleworth *et al*. 2021; Sumner *et al*. 2023). Our first hypothesis is therefore:

Hypothesis 4: Openness is negatively correlated with Brexit support.

Higher levels of openness have also been consistently related to more positive attitudes towards outgroup-size, with a meta-analysis finding that the magnitude of the correlation is substantial (Sibley and Duckitt 2008). Jackson and Poulsen (2005) for example find that individuals high in openness and agreeableness tend to be more likely to initiate contact with ethnic minority group members. This is likely due to individuals scoring higher in openness, when adapting to changing contexts and moving to more diverse environments, being able to take advantage of new environmental encounters. Moreover, Danckert *et al*. (2017) find that individuals scoring higher in openness tend to hold more pro-immigration attitudes as a result of greater intergroup contact as well as self-reported exposure to outgroup-size in their neighbourhood. Evidence looking at openness in the context of neighbourhood outgroup-size stresses both how individuals characterised by high openness tend to be more curious about differences (Sibley and Duckitt 2008; Peresman 2023), which might increase the likelihood of intra-group interactions in the context of higher levels of outgroup-size, but also more novelty-seeking (Phelps *et al*. 2011; Harris and Vazire 2016; Danckert *et al*. 2017), which might drive more positive reactions concerning the rate of increase in neighbourhood outgroup-size. Based on this and on previous evidence connecting openness with political attitudes and behaviours, we thus hypothesise that:

Hypothesis 4a: Higher openness boosts the (negative) relationship between levels of outgroup-size and support for Brexit.

Hypothesis 4b: Higher openness attenuates the (positive) relationship between increases of outgroup-size and greater support for Brexit.

Regarding the remaining three Big Five traits, previous findings are less consistent. Overall, there is no clear evidence on the extent to which conscientiousness, extraversion and emotional stability are correlated with anti-immigrant sentiment (Sibley and Duckitt 2008; Dinesen *et al*. 2016; Gallego and Pardos-Prado 2014).

Research in the field of political psychology has identified a correlation between conscientiousness and conservative attitudes and behaviours. Multiple studies, including those by Mondak and Halperin (2008), Gerber *et al*. (2010), and Mondak (2010), have shown that individuals with higher levels of conscientiousness tend to hold more conservative viewpoints. This suggests a relationship between personality traits and political ideologies. Furthermore, specific studies have delved into the association between conscientiousness and support for Brexit. Schoen and Schumann (2007), Bakker and De Vreese (2016), and Lee *et al*. (2018) have highlighted that individuals demonstrating elevated conscientiousness levels were more inclined to support the Brexit movement. Conscientiousness is a personality trait characterized by organization, discipline, diligence, and a strong sense of duty, and individuals high in conscientiousness tend to be concerned with maintaining order and following established norms. Research has also shown some evidence that conscientiousness can be related with right-wing authoritarianism (Duckitt and Sibley 2014). In the context of the Brexit referendum, this aspect may have made these individuals more inclined to support Brexit on various aspects such as the economy, immigration policies, trade agreements, and national sovereignty. Moreover, conscientious individuals are often more risk-averse and value stability and order. They may have viewed leaving the EU as a way to regain control and create a more structured and self-determined future. In fact, as stressed by Nijs *et al* (2021), many voters and politicians perceived the Brexit referendum as an opportunity to regain control over ‘what is ours’’. Since conscientious individuals typically also place a high value on fulfilling responsibilities and commitments, they may also have seen Brexit as a duty or responsibility to the nation, driven by a sense of patriotism or the belief that it was necessary to prioritize the interests of their country over the benefits of EU membership. Based on these considerations, and the findings of prior research, we therefore propose Hypothesis 5.

Hypothesis 5: Conscientiousness is positively correlated with Brexit support.

Concerning the interaction between personality and neighbourhood outgroup-size, drawing on the threat hypothesis, most authors have suggested that individuals scoring high in conscientiousness tend to react negatively to economic threats deriving from greater ethnic outgroup-size (Hodson *et al*. 2009; Dinesen *et al*. 2016). Ackermann and Ackermann (2015) also find conscientiousness to be related with less favourable attitudes towards immigrants. However, they note that at higher levels of outgroup-size, which favours inter-ethnic contact, the traditionalist attitudes of conscientious individuals are mitigated. Nonetheless, we overall expect:

Hypothesis 5a: Higher conscientiousness attenuates the (negative) relationship between levels of outgroup-size and support for Brexit.

Hypothesis 5b: Higher conscientiousness boosts the (positive) relationship between increases of outgroup-size and greater support for Brexit.

Lee *et al*. (2018) and Areal (2021) find that being extraverted, acting with self-confidence and outspokenness (i.e. agency) is positively related with greater support for Brexit. However, due to their sociability, individuals scoring high in extraversion may be expected to have greater contact with foreigners or outgroups in their country, and thus less anti-immigrant sentiment, although previous research has failed to find consistent empirical support for this conjecture (Ackermann and Ackermann 2015). Phelps *et al*. (2011) find that extraverted individuals tend to have more positive attitudes towards immigrants and are more open to diversity and multiculturalism. Their outgoing and social nature may facilitate interactions and positive experiences with people from different backgrounds, leading to greater acceptance. Based on this:

Hypothesis 6: Extraversion is negatively correlated with Brexit support.

Hypothesis 6a: Higher extraversion boosts the (negative) relationship between levels of outgroup-size and support for Brexit.

Peresman (2023) also finds that, in the UK, as immigrant levels increase in one’s local area, individuals scoring high in extraversion have lower anti-immigrant sentiment towards the immigrant population. However, as also stressed by Peresman (2023) the negative relationship between extraversion and anti-immigrant sentiment towards the ethnic outgroup is significant only at higher immigrant levels, while it is not significant at average values. Hence,

Hypothesis 6b: Higher extraversion attenuates the (positive) relationship between increases of outgroup-size and greater support for Brexit.

Finally, emotional stability is less frequently associated with anti-immigrant sentiment (Freitag and Rapp 2015). Emotional stability has indeed been found to predict more stability in existing and well-known relationships (Harris and Vazire 2016) and when it comes to anti-immigrant sentiment, we thus hypothesise that greater emotional stability will be associated with less anti-immigrant sentiment. Therefore, even if, in the context of the Brexit referendum, Sumner et al. (2023) found that higher emotional stability associates with a Leave position, we hypothesise that:

Hypothesis 7: Emotional stability is negatively correlated with Brexit support.

In particular, individuals scoring higher on this trait may exhibit greater tolerance and adaptability towards immigrants due to their ability to manage and regulate their own emotions effectively. They may be less likely to experience fear, anxiety, or threat in the face of cultural diversity, leading to less anti-immigrant sentiment at higher levels of outgroup-size (Phelps *et al* 2011). In fact, individuals scoring low on emotional stability may easily become anxious, nervous, or troubled (Mondak 2010) and Marcus *et al*. (1995) argue that they might be more likely to perceive immigrants as a threat, with this possibly exacerbating at higher rate of increase in outgroup-size. Accordingly, our final hypotheses are:

Hypothesis 7a: Higher emotional stability boosts the (positive) relationship between increases of outgroup-size and greater support for Brexit.

Hypothesis 7b: Higher emotional stability attenuates the (positive) relationship between increases of outgroup-size and greater support for Brexit.

In summary, we predict that personality traits heterogeneously moderate the relationship between levels and change of outgroup-size and political behaviours connected to immigration in the context of the Brexit referendum (see Table 1 for an overview).

**Table 1.** Overview of the role of each personality trait Brexit support.

		Moderating role	
	Support for Brexit	Levels	Increase
Agreeableness	-	+	-
Openness to Experience	-	+	-
Conscientiousness	+	-	+
Extraversion	-	+	-
Emotional Stability	-	+	-

Note:(+) signals boosting, i.e. increasing the (positive or negative) main effect of outgroup-size on support for Brexit, (-) marks attenuating, i.e. reducing the (positive or negative) main effect of outgroup-size on support for Brexit.

While positioning these hypotheses and this work within the framework of the current debate, one aspect that it is critical to keep in mind is that prior work has mainly considered measures that capture self-reported attitudes, with a relative scarcity of research exploring policy-related behavioural responses to outgroup-size. Understanding the relationship between levels of and changes in outgroup-size, and personality traits on policy-related behaviours in general and, specifically, in the Brexit referendum, is important as such behavioural measures tend to be more impactful than self-reported attitudes. Our study thus adds to and critically extends prior research as it (1) examines both contextual-level (i.e. outgroup-size) and individual-level (i.e. personality traits) factors that drive policy-related behaviours, (2) considers a policy-issue manifestation of anti-immigrant sentiment with critical consequences for society (i.e. the Brexit vote), and (3) considers both levels of and increase in outgroup-size at the neighbourhood level, as well as their interactions with personality traits in explaining Brexit support.

## Data and methods

To address our research question, we exploit data from England and Wales from the Understanding Society survey, which is the largest longitudinal household panel study in the UK (University of Essex 2021). We focus on England and Wales because the dynamics of Brexit and immigration were much more salient there compared to Scotland, where the link between immigration narratives and Brexit was far less strong (Gawlewicz 2020), and Northern Ireland, where inter-ethnic dynamics are more structured by the history of the troubles and unionist/nationalist sentiment (Henderson *et al*. 2017) and where immigration is much lower. All survey procedures were approved by the Ethics Committee of the University of Essex. We use Understanding Society in combination with data on neighbourhood outgroup-size available from Census Statistics for England and Wales. Such data, detailing the demographic composition of respondents’ local areas (Lower Super Output Areas – LSOAs), are retrieved from the 2001 and 2011 census and linked to respondents from Understanding Society.

Since we are interested in investigating individuals’ experiences of outgroup-size in an immediate social environment, we define the neighbourhood at the LSOA level. LSOAs are similar in size, they have stable boundaries (unlike electoral wards) and they are consistent with units used by the Census. LSOAs typically contain between 1000 and 3000 people with an average population of 1,400 people (Manley 2021).

Understanding Society covers information from 1991 to 2017 but we restrict our analyses to white British present in wave 8 of Understanding Society (N = 23,391), which covers the period 2016–2017 and it is the time in which our outcome variable was asked^2^, and we use previous waves to account for retrospective information. We include in our analysis only the white-British majority population, given that perceptions about outgroup-size are typically a more salient issue for the incumbent receiving majority group. We further restrict the sample to those individuals who stayed in the same neighbourhood since at least 2005. By focusing on stayers, we aim to isolate the impact of changes in outgroup-size stemming solely from shifting demographics which occur around an individual who remained in the same community; thus, removing any impact of changes in outgroup-size stemming from individuals moving between communities. The 2005 threshold is motivated by balancing sample size needs and allowing individuals to have enough exposure to changes in neighbourhood ethnic composition between 2001 and 2011.^3^

Our final sample with non-missing responses on the dependent variable and on personality traits is 7,681 individual observations. Most of the sample supported Leave, rather than Remain (52.5 vs. 47.5%), in accordance with official statistics (The Electoral Commission 2021).^4^

## Measures

### Outcome variables

Our outcome variable draws on the question ‘Should the UK remain a member of the EU?’ with possible answers ‘Remain’ or ‘Leave’. We coded as 1 those responses where individuals choose a preference to leave the EU and as 0 those who supported the remain option. While this variable does not measure how the respondents actually voted in the referendum, it measures their support about the UK’s EU membership on the day they were interviewed. However, as people’s views about the European Union are probably highly correlated with their vote in the referendum (if they did vote), the associations reported in this paper are relevant to understanding the social basis of Brexit (Chan *et al*. 2020). Furthermore, as outlined above, when testing the effects of community outgroup-size, their impact on this indicator of support for remaining or leaving the EU can be thought of as strongly denoting their impact on anti-immigrant sentiment.

### Neighbourhood outgroup-size

To capture the level of outgroup-size within a respondent’s residential area we use the proportion of non-white British individuals living in their LSOA. To better engage with the existing literature, it is important to note that, at least in the UK, ethnic outgroup-size tends to correlate very highly with other measures of diversity, such as ethnic fractionalization indexes/the Simpson’s diversity index (Laurence *et al*. 2019) and results tend to be comparable across these different measures. We include both the 2011 percentage levels of non-white British and the increase in LSOA outgroup-size by subtracting, for each LSOA, the proportion of non-white British in 2011 from the proportion of non-white British in 2001 and define this as the percentage increase in non-white British. This approach helps us to overcome the fact that we do not have two different observations for our outcome variable. Therefore, using the difference between 2011 and 2001 (with reference to the outcome measured in 2016) allows us to introduce a dynamic dimension also in the analysis.

### Personality traits

With respect to personality traits, they are measured in 2011 and they are expected to be relatively stable over time (McCrae and Costa 1985). Measures come from a validated scale designed to measure the Big-5 along the five key dimensions: extraversion, agreeableness, conscientiousness, emotional stability, and openness to experience. In 2011 Respondents completed a short, 15-item version of the Big Five Inventory (BFI) originally developed for use in the German Socio-Economic Panel (Schupp and Gerlitz 2014).^5^

### Covariates

At the individual-level, we adjust all our models for participants’ age, gender, tenure, marital status, whether there are children living in the household, employment status, socio-economic group, and educational qualifications. At the LSOA-level, we also adjust our models for the level of urbanisation (a dummy rural/urban), the level of resource-disadvantage in a community (% female headed lone-parent households, % unemployed, % in social housing), and the percentage of people above 65 years old. We additionally control for macro-region. To avoid that missingness in the covariates affect our empirical analysis, all the categorical covariates include a category to account for missingness; the continuous variables included (age, resource-disadvantage, percentage of people above 65 years old) do not have any missing.

### Analytic strategy

Given the cross-sectional structure of the data we estimate different logistic regression models with support for Brexit as the dependent variable. We estimate the model using sampling weights (Kaminska 2021) and robust standard errors clustered at the LSOA level. Our decision of clustering at the LSOA level is motivated by the fact that, as we identify the neighbourhood at the LSOA level we want to avoid within-cluster correlation biases at the treatment level. In all models we consider contemporaneously all 5 personality traits and, besides the covariates for individual and contextual characteristics, we begin by including both the 2011 levels of outgroup-size and increase in outgroup-size between 2001 and 2011. We proceed by including five interactions between each personality trait and the 2011 levels of outgroup-size. We conclude by replacing the latter interactions with five new ones between the personality traits and the increase in outgroup-size between 2001 and 2011.

## Results

We present here the results in terms of odds ratios which can be understood as the probability of the event (i.e. supporting Brexit) divided by the probability that the event does not occur (i.e. supporting Remain).^6^

In Table 2, we see that the 2011 levels of outgroup-size and its increase from 2001 to 2011 have an opposite correlation with the probability of supporting Brexit in the 2016 Brexit referendum, confirming our first two hypotheses. While, indeed, people who were living in a neighbourhood 1 standard deviation greater in terms of outgroup-size had been 1.27 (1/0.782) times less likely to support Brexit, those living in a neighbourhood which experienced a 1 standard deviation larger increase of outgroup-size between 2001 and 2011 had been 1.17 more likely to support Brexit. In terms of size of these effects, when looking at the levels of outgroup-size, the odds of supporting Brexit are 8.4 times lower (standardised levels ranging from 0.77–6.42) for those individuals living in more diverse areas than individuals living in less diverse ones. Conversely, the odds of supporting Brexit for individuals living in areas which experienced the largest outgroup-size increase are 14.6 times higher (standardised change ranging from −3.06 to 8.46) than the odds of those living in the areas which experienced the lowest increase.

**Table 2.** Pooled logistic regression of the impact of 2011 levels non-white British and of % increase (2001-2011) of non-white British population in the residential neighbourhood (LSOA) on Brexit support by Big 5 personality inventory.

	(1)	(2)	(3)
VARIABLES	No interactions	Interactions with levels	Interactions with increase
Outgroup-size (increase)	1.166**	1.149*	0.843
	(0.069)	(0.069)	(0.160)
Outgroup-size (level)	0.782***	0.651*	0.776***
	(0.052)	(0.142)	(0.052)
Agreeableness	0.903***	0.899***	0.900***
	(0.026)	(0.026)	(0.026)
Outgroup-size #Agreableness		1.064*	1.049+
		(0.029)	(0.026)
Extraversion	1.027	1.025	1.026
	(0.021)	(0.021)	(0.021)
Outgroup-size #Extraversion		1.028	1.003
		(0.023)	(0.021)
Conscientiousness	1.092***	1.094***	1.090***
	(0.029)	(0.029)	(0.028)
Outgroup-size #Consciousness		0.985	1.005
		(0.026)	(0.026)
Emotional Stability	1.050*	1.047*	1.049*
	(0.021)	(0.020)	(0.020)
Outgroup-size #Neurotiscism		1.015	1.030
		(0.021)	(0.020)
Openness	0.932**	0.938**	0.934**
	(0.021)	(0.021)	(0.021)
Outgroup-size #Openness		0.942**	0.975+
		(0.020)	(0.021)
			
Constant	1.212	1.211	1.261
	(0.402)	(0.400)	(0.416)
Observations	7,681	7,681	7,681
Pseudo R2	0.071	0.073	0.072

Notes: Authors’ computation on Wave 8 of Understanding Society. Robust standard errors in parentheses. *** p < 0.001, ** p < 0.01, * p < 0.05, +p < 0.1. Weighted results. Full results in the Appendix A.2, table A3.

When we look at personality traits, on average more agreeable and more open individuals are less likely to support Brexit, confirming hypothesis 3 and 4. Extraversion is not significantly associated with Brexit support, so we fail to find evidence for hypothesis 6. Individuals scoring higher in conscientiousness and emotional stability are instead more likely to support Brexit, which is consistent with hypothesis 5 and runs against hypothesis 7.

When we introduce interactions in columns 2 (with levels) and 3 (with change), we see that, first, the previously highlighted findings remain almost identical and, second, the interaction-terms display some heterogeneity according to the different personality traits, but they tend to follow the same direction both with the levels and the increase. Crucially, the only two interaction terms that reach statistical significance are those between levels, or increase, in outgroup-size and, respectively, agreeableness and openness (i.e. hypotheses 5a/b, 6a/b, and 7a/b are not supported). Looking at the former, the coefficient suggests that the gap in the probability of supporting Brexit between those scoring high or low on agreeableness closes as outgroup-size increases. Conversely, with respect to openness, we see that the interaction term goes in the opposite direction, suggesting that an increase in outgroup-size goes hand in hand with a reduction of the gap in the probability of supporting Brexit between those scoring high or low on openness.

**Figure 1.**
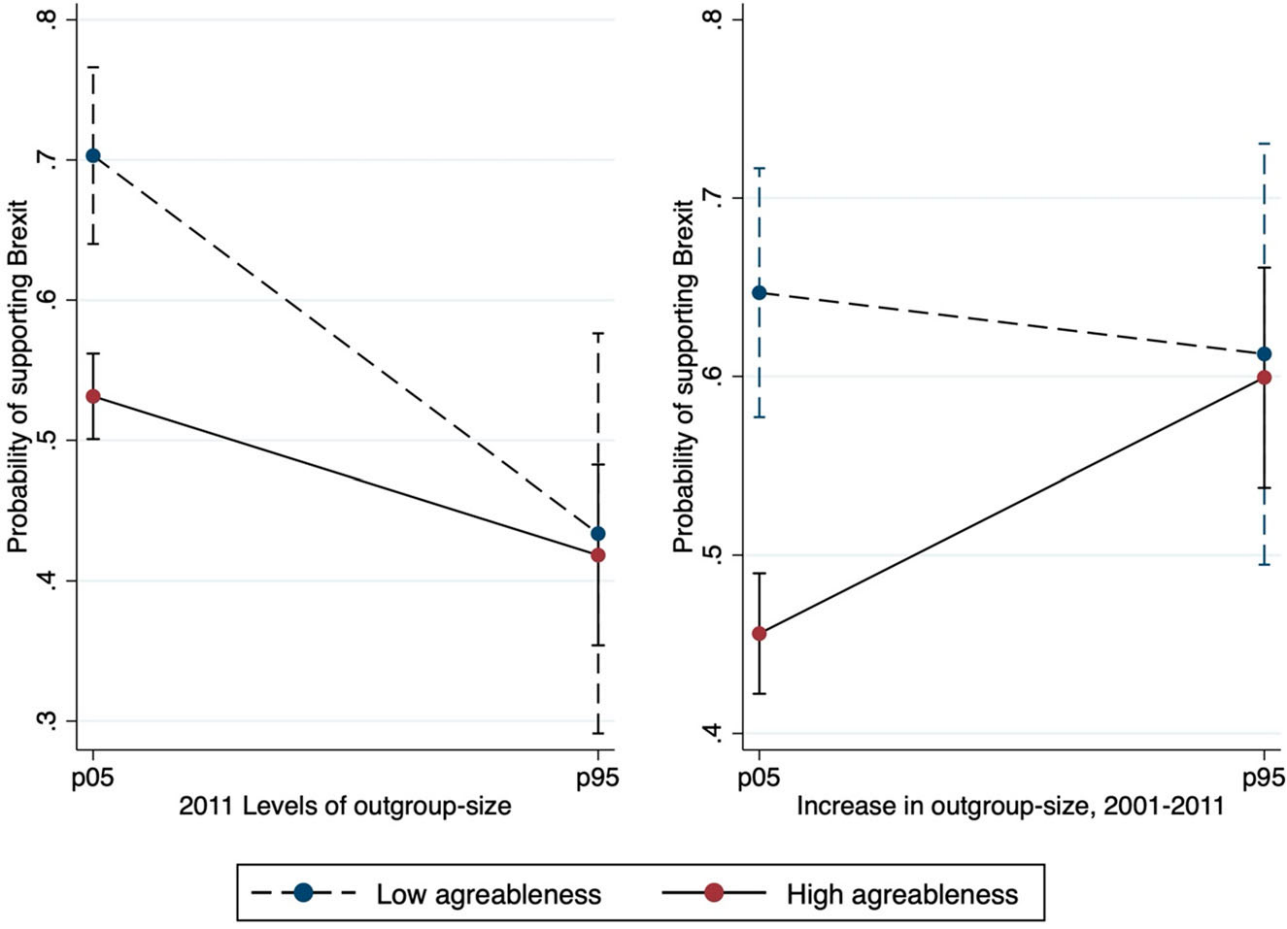
Predicted probabilities of Brexit support across levels and % increase in non-white British by level of agreeableness, predictive margins with 95% Cis. Notes: Authors’ computation on Wave 8 of Understanding Society dataset. Graph are plotted at minimum and maximum values of agreeableness (1, 7) and at the 5^th^ and 95^th^ percentile of the distributions of outgroup-size levels (−0.65, 2.09) and increase (−0.82, 2.07). Weighted results.

**Figure 2.**
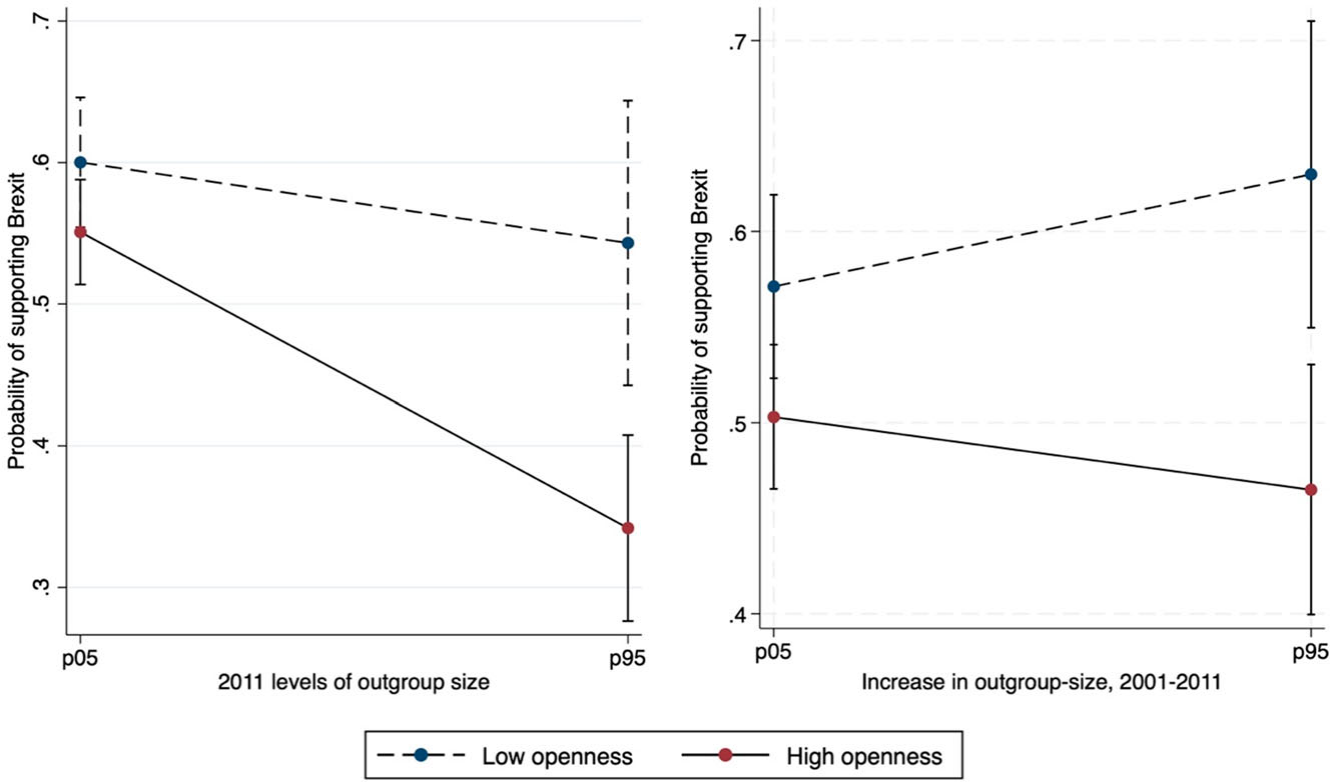
. Predicted probabilities of Brexit support across levels and % increase in non-white British by level of openness, predictive margins with 95% Cis. Notes: Authors’ computation on Wave 8 of Understanding Society dataset. Graph are plotted at minimum and maximum values of openness (1, 7) and at the 5<suthp> and 95<suthp> percentile of the distributions of outgroup-size levels (−0.65, 2.09) and increase (−0.82, 2.07). Weighted results.

Figures 1 and 2 visualize the interaction coefficients for agreeableness and openness, respectively presented in Table 2 (columns 2 and 3).^7^ In both cases, there is an overall downward trend when considering the 2011 levels and a predominantly upward trend when considering the increase in outgroup-size between 2001 and 2011.

Figure 1 shows that, for low outgroup-size scores (both levels and increase) people scoring higher in agreeableness are significantly less likely to support Brexit than people scoring low. The greater the levels of outgroup-size, greater agreeableness does not boost the effect of the contextual variable on Brexit support, which runs against our hypothesis 3a. In fact, it is just at lower levels of outgroup-size that we observe that being more agreeable significantly reduces the probability of supporting Brexit. At the same time, greater agreeableness also does not seem to attenuate, while actually boosting, the positive relationship between increases in outgroup-size and Brexit support, failing to support hypothesis 3b. We find indeed evidence for an opposite pattern to what we had expected. High agreeable individuals are the ones who showcase a marked increase in Brexit support the greater the increase in outgroup-size.

Figure 2 shows the pattern for openness. In this case, for levels, coherently with hypothesis 4a, we observe that greater openness boosts the effect of greater outgroup-size on the (lower) probability of supporting Brexit. In contrast with agreeableness, results show that openness matters especially at higher levels of outgroup-size. When looking at the increase in outgroup-size, we find support for hypothesis 4b, for which openness attenuates (although marginally) the positive effect of this contextual variable on Brexit support. In fact, while at low levels of increase in outgroup size openness does play a significant role, at higher levels of outgroup-size increase, the gap in the probability of supporting Brexit between those scoring high vs low in openness is significant. It thus suggests that the widening of the gap is significant because the patterns in Brexit support of those scoring low vs high in openness diverge as the increase in outgroup size becomes greater.

## Discussion and conclusions

In this work, we have investigated the trilateral relationship between (a) levels and increasing outgroup-size in the neighbourhood of residence, (b) individuals’ personality traits and (c) policy-related responses to outgroup-size. We have used the Understanding Society survey, where, participants were asked whether they were more likely to support Leave or Remain in the 2016 Brexit referendum. We demonstrated that this measure of support for leaving the EU acts as a good proxy for capturing anti-immigrant sentiment when examining the effects of ethnic outgroup-size. Insofar as respondents’ preferences towards leaving the EU map closely on to their actual voting behaviour, the measure used in this study has actual measurable consequences (which more general attitudinal measures cannot provide us with), as it ties also to concerns about nation-wide trends of immigration in the UK (although of course, as discussed, the issue of immigration is not the only reason that determined people’s voting for Brexit).

Specifically, we examined the extent to which the relationship between changing neighbourhood outgroup-size and policy views related to anti-immigrant sentiment is moderated by differences in people’s personality traits. There are four main findings of this paper: first, the higher the levels of outgroup-size in 2011, the lower the support for Brexit (confirming hypothesis 1); second, the higher the increase in outgroup-size from 2001 to 2011, the higher the support for Brexit (confirming hypothesis 2); third, people scoring higher in agreeableness and openness were less likely to support Brexit (confirming hypotheses 3 and 4), people scoring higher in conscientiousness and emotional stability were more likely to support Brexit (confirming hypothesis 5 and failing to confirm hypothesis 7), no association is found between extraversion and support for Brexit (failing to confirm hypothesis 6); fourth, as outgroup-size increases, the gap in the probability of supporting Brexit, between people scoring high and low in agreeableness (openness) decreases (increases), therefore contradicting hypotheses 3a/b and confirming hypotheses 4a/b (while we do not find support for hypotheses 5a/b, 6a/b, 7a/b). Our analyses lead us to conclude that agreeableness and openness are the most important traits when assessing how changing outgroup-size at the local level may affect individuals’ intended political behaviours in relation to the impacts of outgroup-size. However, two interesting elements deriving from our results are that levels of outgroup-size and the increase in outgroup-size have opposite patterns. While at increasing outgroup-size rates the difference in the outcome between more or less agreeable individuals diminishes, it instead augments between more or less open individuals.

Our results concerning the role of levels and change appear to be consistent with theories of intergroup contact. Individuals living in areas characterised by high levels of outgroup-size may have more opportunities to engage in positive inter-ethnic contact. It is reasonable to assume that it may require a greater effort to interact with people from different ethnic backgrounds in areas where the latter are a smaller minority than in areas where they represent a larger group. The fact that on average both openness and agreeableness have a positive correlation with the outcome is, again, consistent with intergroup contact theories, and is also in line with prior work by Danckert *et al*. (2017). In fact, for both traits, we find an average negative correlation with the probability to support Brexit and that both people scoring high and low in the respective scales, are less likely to prefer Brexit in areas characterised by high levels of outgroup-size. Moreover, for openness we observe a general widening of the gap between people scoring low and high on this trait, which again is coherent with contact theory. This pattern is more evident when we consider levels of outgroup-size than when we consider the increase thereof. However, for agreeableness, those scoring low are much more likely to support Brexit than people scoring high when living in an environment characterised by lower levels of diversity, but such likelihood decreases much more steeply for them than for individuals scoring high. When we look at the increase of outgroup-size, we even see that larger increases in outgroup-size make high agreeable individuals as likely to support Brexit as individuals scoring low on the same trait, almost reversing the pattern we see when the increases are more moderate.

A possible explanation for this can be traced to research on personality and political behaviour. This literature posits, on the one hand that more agreeable individuals tend to be keener to avoid confrontation and to take on political positions that seem more conventional and widely shared by the majority (Gerber *et al*. 2010). As stated by Mondak and Halperin (2008): ‘the confrontational aspects of politics are disconcerting to individuals with high levels of agreeableness’ (346). On the other hand, openness has been commonly associated with more liberal approaches and, if anything, people scoring high in openness are expected to be ‘ … relatively interested in and attentive to politics*’* (Mondak and Halperin 2008: 342).

High agreeable individuals residing in neighbourhoods experiencing rapid increases in outgroup-size (where the increase in immigration was clearly apparent and more salient), may have experienced the Brexit debate as more divisive and conflicting than their counterparts living in neighbourhoods characterised by lower increases. Since the probability to support Brexit is larger in areas which experienced greater increases in outgroup-size, more agreeable individuals living in such areas, might have been more willing to conform to the local predominant norm and support Brexit themselves, to avoid confrontation. Thus, when confronted with such a divisive theme, they might have become more likely to support Brexit conforming to voting preference prevalent in their area (resulting in the steeper increase trend in Figure 1, right panel). Looking at this from the angle of social conservatism, engaging in behaviours such as expressing voting preferences that have a real and concrete impact could be more costly for individuals who significantly care about prevalent social norms than just expressing personal opinions and preferences. Similarly, while being exposed to greater levels of outgroup-size may have lowered the chances of people who score low in agreeableness to support Brexit (steeper decrease in Figure 1, left panel) this may not have had a big impact on people scoring high on the same trait. Conversely, with respect to openness, people scoring high might have experienced an additive effect of living in areas characterised by higher outgroup-size levels, which may have led them to be even more engaged into politics and more committed to defend the opportunities provided by remaining in the EU (steeper decrease in Figure 2, left panel). The same interest in and commitment to politics may have reduced their increased support to Brexit when experiencing larger changes in outgroup-size, a phenomenon that conversely may have amplified the increase of people scoring low in openness (steeper increase in Figure 2, right panel). To sum up, outgroup-size seems to have played a levelling effect on how individual differences in agreeableness affect Brexit support. In contrast, it has emphasised individual differences with respect to openness.

Notably, our findings diverge from those of Silva *et al*. (2023), which focused exclusively on attitudinal measures of social distance (i.e. prejudicial attitudes). Silva *et al*. (2023) demonstrate that agreeableness plays the most important role in moderating the effects of outgroup-size on attitudes towards immigrants. Positive changes in outgroup-size had little association with prejudice among more agreeable individuals but showed increased prejudice levels among less agreeable individuals. Our research here examined intended behavioural measures of Brexit support, and although we found that agreeableness played a key role, we also (and unlike Silva *et al*. 2023) observed that openness matters. In addition, we found that low agreeableness individuals are more likely to support Brexit, which corresponds to Silva *et al*.’s (2023) finding that such individuals have higher levels of prejudice. However, we also find that increasing outgroup-size has no relationship with Brexit support among people scoring low in agreeableness because they have high levels of Brexit support regardless of the amount of outgroup-size change in their communities. Instead, increasing outgroup-size has a positive effect on Brexit support among high agreeable individuals, who report low Brexit support in areas not experiencing outgroup-size change, but whose Brexit support increases as the amount of outgroup-size change increases. We attribute this discrepancy to the fundamental distinction in the two outcome variables; indeed the more social conventional nature of high agreeable individuals explained above, while not reflected in attitudes, it becomes evident on political behaviours.

Therefore, the current study provides novel, complementary insights to work such as Silva *et al*.’s (2023) into how increasing outgroup-size and personality are related to manifestations of anti-immigrant sentiment, by showing that its impact on behavioural manifestations can differ from its attitudinal manifestations. Future research needs to keep this in mind, and consider both attitudes and behaviours for understanding the wider impact of diversity on societies.

Notwithstanding the contributions of our paper, our results do not warrant causal conclusions to be drawn on the role of increasing neighbourhood outgroup-size on ethnic-related preferences. To try to limit issues related to residential selection, we followed previous research (Laurence and Bentley 2016) and focused on stayers (to keep changing outgroup-size as exogenous as possible). Nonetheless, there remains the potential impact of residential selection prior to the 2000s. Similarly, we were only able to use information from the 2011 census as the most recent 2021 census data has not yet been released. Given the continuous change in outgroup-size, it would be interesting for future research to further explore the dynamics we have investigated once the updated census statistics from the 2021 census will be released. Another possibility of making our findings more robust, could be to consider longer time periods, focusing jointly on movers as well as stayers and possibly creating a cumulative measure of exposure to outgroup-size across the life course. Further research should also seek to address some additional limitations of our study. Firstly, we provided strong evidence that, at least when it comes to the effect of outgroup-size, support for leaving the EU likely acts as a key manifestation of anti-immigrant sentiment. However, future research may also test whether the results found in the current study also hold in other contexts across the world and/or considering other policy-related measures. Secondly, we demonstrated that Brexit support was sensitive to the share of non-white British in individuals’ community, the impact of which was moderated by personality traits. However, research has suggested Brexit support may have been particularly sensitive to certain forms of migration, such as Eastern Europeans (Martin, Sobolewska and Begum 2019). Accordingly, future research could explore how far the moderating role of personality might differ for proximity to (and changes in) different ethnic outgroups in an area, beyond all non-white British alone.

To conclude, our paper provides compelling evidence on how outgroup-size in neighbourhoods was related to Brexit support and how these effects were further shaped by two key personality traits, agreeableness and openness. Our analyses thus show the complex and intertwined nature of both contextual – and individual – level factors, and how this can critically shape political behaviours.

## Supplementary Material

Supplemental Material

## Footnotes

1 In this paper, to avoid repetitions, by using outgroup-size we specifically mean community (neighbourhood) ethnic outgroup-size. Other uses of the word will be clearly stated.

2 We use information from previous waves from the BHPS to retrieve information on personality and on where individuals were living before the observation period.

3 We tried with a 2001 threshold and the results remain the same, although with a smaller sample size.

4 Table A0 in the appendix show descriptive statistics about the full sample of white-British in wave 26, the sample of stayers, and the empirical sample on which our results is based. From this table it can be appreciated that the empirical sample does not differ very much from the whole sample, especially with respect to personality traits and diversity.

5 Each trait is built upon three sub-indicators, based on items such as ‘Active imagination’ (openness to experience), ‘Kind’ (agreeableness), ‘Is sociable’(extraversion), ‘Efficient’(conscientiousness) and ‘Worries a lot’ (emotional stability).

6 In order to interpret the odds ratios in terms of probability, the odds ratios can be divided by (1+odds ratio)

7 Since we are actually interested in understanding how the pattern evolves at extreme values we plot at p5 and p95. Nonetheless, we have plotted the same at p10/90 and p20/80. The results do not change, they are just less pronounced.
