# Supplementary material for: Does personality matter? Exploring its moderating role on the relationship between neighbourhood ethnic outgroup-size and preferences for Brexit
Source: Eur Soc. 2023 Nov 6;26(4):1012–45. doi: 10.1080/14616696.2023.2277279 (PMC11378495; doi:10.1080/14616696.2023.2277279)
Supplement: Supplemental Material [file REUS_A_2277279_SM6126.docx]

**Appendix A.1 – Testing the relationship between ethnic composition/ethnic change and voting leave via their association with anti-immigrant sentiment.**

The following analysis seeks to establish evidence that the association between our indicators of the ethnic composition of people’s residential communities (proportion non-White British (2011) and the change in the proportion non-White British (2001-2011)) and support for voting to leave the EU is likely to primarily come through the effects of community ethnic composition on attitudes towards immigration. In other words, while support for Brexit had multiple drivers (of which anti-immigrant sentiment was only one), when it comes to the effects of community ethnic composition on support for Brexit, the primary driver was via its effect on anti-immigrant sentiment.

To investigate this question, we turn to another dataset: the 2017 British Election Study (BES). The BES contains measures of both support for leaving the EU (‘Thinking back to the EU referendum…, if you voted did you vote to remain in the EU or to leave the EU, or did you not vote?’) and attitudes towards immigration (‘Do you think that too many immigrants have been let into this country, or not?’ and ‘How strongly do you feel about this?’). Our dependent variable therefore is voted to leave the EU coded as 1 (with voted to remain coded as 0)^[[1]](#footnote-1)^. Our measure of immigration attitudes is coded from (1) ‘Not too many immigrants/feel very strongly about this’, (2) ‘Not too many immigrants/feel fairly strongly’, (3) ‘Not too many immigrants/feel not very strongly’, (4) ‘Too many immigrants/feel not very strongly’, (5) ‘Too many immigrants/feel fairly strongly’, and (6) ‘Too many immigrants/feel very strongly about this’. We also use the same measures of ethnic composition applied in the remainder of the article: diversity is captured using ‘proportion non-White British’ in 2011 (coded 0 to 1, where 1=100%^[[2]](#footnote-2)^) and change in diversity is captured using ‘change in the proportion non-White British between 2001 and 2011’ (coded -1 to 1, where 1=change from 0% to 100%). Where possible, we sought to replicate the remaining individual- and community-level covariates applied in the rest of the paper. At the individual-level, our BES analysis therefore includes age, gender, education, employment status, tenure, martial status, perceived financial situation, social class and an indicator of which survey company collected which responses. At the community-level, we include a measure of resource disadvantage (per cent female headed lone-parent households, per cent unemployed, per cent in social housing) and urban/rural location. We also restrict the sample to White British respondents. One notable differences is that the BES only allows us to test these relationships at the Middle Super Output Area level (not the LSOA-level as applied in the remainder of the paper).

To test how far the relationships between diversity/change in diversity and support to leave the EU are driven by immigration attitudes we perform three models. Model 1 (multilevel logistic regression) tests the associations between diversity/change in diversity and support to leave the EU. Model 2 (multilevel mixed effects linear regression) tests the associations between diversity/change in diversity and strength of feeling that there are ‘too many immigrants let into this country’. Model 3 (multilevel logistic regression) tests the associations between diversity/change in diversity and support to leave the EU, after accounting for immigration attitudes. Given the use of logistic regressions, all model coefficients are average marginal effects.

Model 1 (Table A.1) finds that, as demonstrated in the paper, there is a negative association between the share of non-White British (2001) in a community and support for leaving the EU, and there is a positive association between change in the share of non-White British (2001-2011) and support for leaving the EU. In Model 2, we examine the associations between diversity and change in diversity and anti-immigrant sentiment. It demonstrates that White British respondents living in areas with a larger share of non-White British report less anti-immigrant sentiment. However, White British respondents living in areas that saw their share of non-White British increase more between 2001 and 2011 report more anti-immigrant sentiment. Finally, Model 3 replicates Model 1 but includes in the model individuals’ anti-immigrant sentiment. We observe a strong positive association between one’s anti-immigrant sentiment and support for leaving the EU. However, more importantly, once we account for anti-immigrant sentiment, the average marginal effect of the level of diversity is reduced by 56 per cent, and is now no longer statistically significant, and the average marginal effect of change in diversity is reduced by 59 per cent and is now no longer statistically significant.

Taken together, this analysis provides strong evidence in support of our paper’s position that a substantial part of the association between a community’s ethnic composition and residents’ propensity to support leaving the EU comes through how the former is linked to residents’ anti-immigrant sentiment. Notably, we find that over 50 per cent of the association is accounted for by this single indicator of immigration attitudes (‘too many immigrants’). Given immigration attitudes are also multiple dimensional, for example, encapsulating further elements of perceived immigrant threat, it might be expected that with further measures of immigration attitudes available to test these relationships would be mediated even further.

**Table A.1 – Relationships diversity/change in diversity, attitudes towards immigrants and support for leaving the EU (2017 British Election Study; average marginal effects)**

|  | Model 1 | Model 2 | Model 3 |
| --- | --- | --- | --- |
| Outcome | Voted leave | Too many immigrants | Voted leave |
| Model type | Logistic | Linear | Logistic |
| Coefficients | AME | AME | AME |
|  |  |  |  |
| *Individual-level* |  |  |  |
| Age | 0.004** | 0.013* | 0.002 |
|  | (0.002) | (0.005) | (0.002) |
| Female | -0.025 | 0.077 | -0.058 |
|  | (0.038) | (0.102) | (0.044) |
| Education: GCSE or lower |  |  |  |
| A-level | -0.033 | -0.319* | 0.041 |
|  | (0.055) | (0.150) | (0.067) |
| Degree or above | -0.169*** | -0.857*** | -0.033 |
|  | (0.041) | (0.135) | (0.053) |
| Employment status: employed |  |  |  |
| Unemployed | -0.220+ | -0.334 | -0.205 |
|  | (0.123) | (0.349) | (0.131) |
| Student | -0.132 | -1.385 | 0.128 |
|  | (0.332) | (1.014) | (0.205) |
| Other employment status | 0.074 | -0.066 | 0.130+ |
|  | (0.063) | (0.211) | (0.070) |
| Retired | 0.013 | 0.149 | -0.018 |
|  | (0.056) | (0.171) | (0.066) |
| Tenure: homeowner |  |  |  |
| rented privately | -0.027 | -0.089 | -0.014 |
|  | (0.063) | (0.182) | (0.074) |
| housing association/local authority | 0.082 | 0.202 | 0.069 |
|  | (0.070) | (0.170) | (0.081) |
| Marital status: non-married |  |  |  |
| married/living with partner | -0.024 | -0.037 | -0.024 |
|  | (0.038) | (0.103) | (0.046) |
| Anticipate financial situation will get better | 0.059* | 0.103 | 0.053+ |
|  | (0.026) | (0.076) | (0.031) |
| Survey agency: GfK |  |  |  |
| Kantar | 0.115* | 0.259+ | 0.091+ |
|  | (0.049) | (0.140) | (0.054) |
| Natcen | 0.052 | 0.120 | 0.041 |
|  | (0.052) | (0.149) | (0.058) |
| Social class: Professional occupation |  |  |  |
| Managerial and Technical occupations | -0.015 | 0.185 | -0.082 |
|  | (0.065) | (0.181) | (0.079) |
| Skilled occupations - non-manual | 0.040 | 0.471* | -0.066 |
|  | (0.078) | (0.207) | (0.090) |
| Skilled occupations - manual | 0.162* | 0.754*** | 0.028 |
|  | (0.077) | (0.226) | (0.092) |
| Partly skilled occupations | 0.194* | 0.897*** | 0.037 |
|  | (0.082) | (0.225) | (0.102) |
| Unskilled occupations | 0.272* | 1.149*** | 0.043 |
|  | (0.136) | (0.288) | (0.153) |
| *MSOA-level* |  |  |  |
| Urban/rural: rural |  |  |  |
| Urban | -0.042 | -0.140 | -0.028 |
|  | (0.045) | (0.120) | (0.054) |
| Community Resource disadvantage (2011) | 0.086** | 0.282*** | 0.053 |
|  | (0.028) | (0.077) | (0.033) |
| % non-White (2011) | -0.628* | -2.213** | -0.276 |
|  | (0.300) | (0.764) | (0.256) |
| Change in % non-White (2001-2011) | 1.610* | 5.910** | 0.653 |
|  | (0.807) | (2.008) | (0.869) |
| Too many immigrants |  |  | 0.207*** |
|  |  |  | (0.016) |
|  |  |  |  |
| Observations | 1129 | 1129 | 1129 |

**Appendix A.2 – Supplementary tables**

**Table A2: Descriptive Statistics, mean(sd)**

|  | (1) | (2) | (3) |
| --- | --- | --- | --- |
|  | Full sample, white people, wave 26 | Sample of interest, stayers | Empirical sample |
| Support for Brexit | 0.490 (0.500) | 0.522 (0.500) | 0.525 (0.499) |
| Increase in % non-white British from 2001 to 2011 | 0.042 (0.055) | 0.040 (0.055) | 0.038 (0.052) |
| % non-white British 2011 | 0.117 (0.145) | 0.113 (0.144) | 0.106 (0.133) |
| Local area deprivation | -0.163 (0.824) | -0.192 (0.824) | -0.241 (0.786) |
| Percentage of people aged >60 in the local area | 0.182 (0.068) | 0.186 (0.066) | 0.188 (0.066) |
| Agreeableness | 5.634 (1.015) | 5.671 (1.003) | 5.664 (1.006) |
| Extraversion | 4.612 (1.309) | 4.578 (1.328) | 4.579 (1.332) |
| Conscientiousness | 5.513 (1.081) | 5.567 (1.076) | 5.566 (1.069) |
| Emotional stability | 4.436 (1.437) | 4.546 (1.431) | 4.557 (1.424) |
| Openness | 4.551 (1.289) | 4.481 (1.304) | 4.501 (1.286) |
| Age | 51.080 (18.750) | 60.027 (15.725) | 61.310 (13.706) |
| Sex, female (dummy) | 0.544 (0.498) | 0.553 (0.497) | 0.561 (0.496) |
| Child in HH: Yes (dummy) | 0.231 (0.422) | 0.129 (0.335) | 0.126 (0.332) |
| Tenure, Own home | 0.744 (0.436) | 0.848 (0.359) | 0.871 (0.335) |
| Tenure, Social Renting | 0.132 (0.338) | 0.109 (0.311) | 0.093 (0.290) |
| Tenure, Private Renting | 0.104 (0.305) | 0.027 (0.163) | 0.024 (0.154) |
| Tenure, Other | 0.002 (0.046) | 0.002 (0.042) | 0.002 (0.044) |
| Marital status, Single/Never Married | 0.194 (0.396) | 0.128 (0.335) | 0.097 (0.296) |
| Marital status, Married/Living as a couple | 0.658 (0.474) | 0.692 (0.462) | 0.718 (0.450) |
| Marital status, Separated | 0.013 (0.112) | 0.010 (0.097) | 0.010 (0.099) |
| Marital status, Divorced | 0.068 (0.252) | 0.073 (0.259) | 0.078 (0.268) |
| Marital status, Widowed | 0.065 (0.246) | 0.096 (0.295) | 0.096 (0.295) |
| Employment status, Unemployed (dummy) | 0.426 (0.494) | 0.524 (0.499) | 0.524 (0.499) |
| Profession, Management & professional | 0.246 (0.430) | 0.191 (0.393) | 0.202 (0.402) |
| Profession, Intermediate | 0.076 (0.265) | 0.065 (0.246) | 0.065 (0.247) |
| Profession, Small employers & own account | 0.058 (0.233) | 0.055 (0.228) | 0.056 (0.231) |
| Profession, Lower supervisory | 0.040 (0.195) | 0.034 (0.180) | 0.030 (0.171) |
| Profession, Semi-routine & routine | 0.144 (0.351) | 0.123 (0.328) | 0.116 (0.320) |
| Education, Low Educ | 0.105 (0.307) | 0.144 (0.351) | 0.126 (0.332) |
| Education, Medium Educ | 0.429 (0.495) | 0.398 (0.490) | 0.388 (0.487) |
| Education, High Educ | 0.371 (0.483) | 0.336 (0.472) | 0.366 (0.482) |
| Education, Other | 0.092 (0.289) | 0.120 (0.325) | 0.119 (0.323) |
| Living in a urban context (dummy) | 0.742 (0.438) | 0.728 (0.445) | 0.722 (0.448) |
| Region, North East | 0.051 (0.220) | 0.048 (0.215) | 0.051 (0.220) |
| Region, North West | 0.125 (0.331) | 0.128 (0.334) | 0.131 (0.338) |
| Region, Yorkshire and Humber | 0.106 (0.308) | 0.103 (0.304) | 0.093 (0.291) |
| Region, East Midlands | 0.094 (0.292) | 0.094 (0.292) | 0.093 (0.291) |
| Region, West Midlands | 0.091 (0.287) | 0.090 (0.286) | 0.090 (0.287) |
| Region, East of England | 0.106 (0.308) | 0.112 (0.315) | 0.116 (0.320) |
| Region, London | 0.064 (0.245) | 0.065 (0.246) | 0.057 (0.232) |
| Region, South East | 0.153 (0.360) | 0.151 (0.358) | 0.155 (0.362) |
| Region, South West | 0.111 (0.314) | 0.099 (0.299) | 0.106 (0.308) |
| Region, Wales | 0.098 (0.297) | 0.110 (0.313) | 0.107 (0.309) |
| Observations | 23,391 | 11,217 | 7,681 |

***Notes:*** Authors’ computation on Wave 8 of the UKHLS dataset. Standard deviation in parentheses. Dummy variables do not have missing observations. Categorical variables which do not sum up to 1 have that fraction as missing. Both diversity measures are standardized (mean 0, standard deviation 1) over the empirical sample.

**Table A3. Pooled logistic regression of the impact of 2011 levels non-white British and of % change (2001-2011) of non-white British population in the residential neighbourhood (LSOA) on Brexit support by Big 5 personality inventory, full table**

| VARIABLES | No interactions | Interactions with change | Interactions with levels |
| --- | --- | --- | --- |
|  |  |  |  |
| Diversity (change) | 1.166** | 0.843 | 1.149* |
|  | (0.069) | (0.160) | (0.069) |
| Diversity (level) | 0.782*** | 0.776*** | 0.651* |
|  | (0.052) | (0.052) | (0.142) |
| Agreeableness | 0.903*** | 0.900*** | 0.899*** |
|  | (0.026) | (0.026) | (0.026) |
| Diversity#Agreableness |  | 1.049+ | 1.064* |
|  |  | (0.026) | (0.029) |
| Extraversion | 1.027 | 1.026 | 1.025 |
|  | (0.021) | (0.021) | (0.021) |
| Diversity#Extraversion |  | 1.003 | 1.028 |
|  |  | (0.021) | (0.023) |
| Conscientiousness | 1.092*** | 1.090*** | 1.094*** |
|  | (0.029) | (0.028) | (0.029) |
| Diversity#Consciousness |  | 1.005 | 0.985 |
|  |  | (0.026) | (0.026) |
| Emotional Stability | 1.050* | 1.049* | 1.047* |
|  | (0.021) | (0.020) | (0.020) |
| Diversity#Neurotiscism |  | 1.030 | 1.015 |
|  |  | (0.020) | (0.021) |
| Openness | 0.932** | 0.934** | 0.938** |
|  | (0.021) | (0.021) | (0.021) |
| Diversity#Openness |  | 0.975+ | 0.942** |
|  |  | (0.021) | (0.020) |
| Age | 1.008* | 1.008* | 1.008* |
|  | (0.003) | (0.003) | (0.003) |
| Sex (female) | 0.829*** | 0.828*** | 0.825*** |
|  | (0.042) | (0.042) | (0.042) |
| Child in HH: Yes | 1.100 | 1.105 | 1.109 |
|  | (0.108) | (0.108) | (0.109) |
| Tenure, Social Renting | 1.427** | 1.435** | 1.425** |
|  | (0.158) | (0.158) | (0.157) |
| Tenure, Private Renting | 1.193 | 1.190 | 1.193 |
|  | (0.222) | (0.222) | (0.224) |
| Tenure, Other | 0.512 | 0.519 | 0.509 |
|  | (0.375) | (0.380) | (0.372) |
| Marital status, Married/Living as a couple | 1.200 | 1.206 | 1.202 |
|  | (0.118) | (0.119) | (0.119) |
| Marital status, Separated | 1.173 | 1.185 | 1.181 |
|  | (0.317) | (0.321) | (0.320) |
| Marital status, Divorced | 1.329* | 1.333* | 1.325* |
|  | (0.178) | (0.178) | (0.176) |
| Marital status, Widowed | 0.937 | 0.940 | 0.936 |
|  | (0.129) | (0.130) | (0.129) |
| Employment status, Unemployed | 1.066 | 1.047 | 1.103 |
|  | (0.409) | (0.390) | (0.395) |
| Profession, Management & professional | 0.559*** | 0.556*** | 0.554*** |
|  | (0.056) | (0.056) | (0.056) |
| Profession, Intermediate | 0.797 | 0.790 | 0.792 |
|  | (0.101) | (0.101) | (0.101) |
| Profession, Small employers & own account | 0.937 | 0.938 | 0.939 |
|  | (0.121) | (0.122) | (0.122) |
| Profession, Lower supervisory | 1.206 | 1.201 | 1.201 |
|  | (0.216) | (0.214) | (0.217) |
| Education, Medium Educ | 0.810* | 0.811* | 0.812* |
|  | (0.076) | (0.076) | (0.077) |
| Education, High Educ | 0.400*** | 0.401*** | 0.401*** |
|  | (0.040) | (0.040) | (0.040) |
| Education, Other | 1.178 | 1.183 | 1.190 |
|  | (0.131) | (0.131) | (0.132) |
| Living in urban area | 1.133 | 1.133 | 1.143 |
|  | (0.079) | (0.079) | (0.080) |
| Region, Nort West | 0.941 | 0.929 | 0.931 |
|  | (0.144) | (0.142) | (0.142) |
| Region, Yorkshire & Humber | 0.923 | 0.914 | 0.922 |
|  | (0.145) | (0.144) | (0.145) |
| Region, East Midlands | 1.102 | 1.090 | 1.093 |
|  | (0.177) | (0.175) | (0.175) |
| Region, West Midlands | 1.112 | 1.102 | 1.105 |
|  | (0.181) | (0.180) | (0.180) |
| Region, East of England | 1.114 | 1.101 | 1.110 |
|  | (0.175) | (0.173) | (0.174) |
| Region, London | 1.261 | 1.255 | 1.290 |
|  | (0.267) | (0.266) | (0.275) |
| Region, South East | 0.922 | 0.915 | 0.920 |
|  | (0.140) | (0.139) | (0.139) |
| Region, South West | 0.988 | 0.978 | 0.980 |
|  | (0.155) | (0.153) | (0.153) |
| Region, Wales | 0.704* | 0.698* | 0.704* |
|  | (0.109) | (0.108) | (0.108) |
| Local area deprivation | 1.030 | 1.027 | 1.026 |
|  | (0.050) | (0.050) | (0.050) |
| Percentage of people aged >60 in the local area | 0.704 | 0.705 | 0.654 |
|  | (0.358) | (0.359) | (0.333) |
| Constant | 1.212 | 1.261 | 1.211 |
|  | (0.402) | (0.416) | (0.400) |
|  |  |  |  |
| Observations | 7,681 | 7,681 | 7,681 |
| Pseudo R2 | 0.0714 | 0.0724 | 0.0732 |

***Notes:*** Authors’ computation on Wave 8 of the UKHLS dataset. Robust standard errors in parentheses. *** p<0.001, ** p<0.01, * p<0.05, +p<0.1. Weighted results. Reference categories: Tenure, Privately Owned; Marital status, Single/Never married; Employment status, employed; Profession, Semi-routine and routine; Education, Low educated; Region: North East.

**Appendix A.3 – Stata code**

*we start from the full bhps-ukhls data harmonised from wave 1-28, downlodable from the UK data archive

*from each wave use the "indresp" file

*merge each wave with LSOA information obtained under special licence

*append all waves

bysort pidp: egen minwave=min(wave)

bysort pidp: egen maxwave=max(wave)

bysort pidp: egen maxrace=max(racel_dv)

keep if wave>11

keep if wave<27

//restricting to England and Wales

keep if gor_dv<11

bysort pidp: gen count=_n

bysort pidp: gen maxcount=_N

//Original sample

gen bhps=2 if memorig>2 & memorig<7

replace bhps=1 if memorig==2 | memorig==1

replace bhps=0 if memorig>6 & memorig!=.

//Creating an indicator to restrict the samples to those who have been in the same LSOA since at least 2005

gen lsoastart= lsoaencoded if count==1

bysort pidp: egen lsoa_start=max(lsoastart)

bysort pidp: egen maxmvyr=max(mvyr)

bysort pidp: egen maxplnowy4=max(plnowy4)

bysort pidp: gen memelsoa=1 if lsoaencoded==lsoa_start

bysort pidp: replace memelsoa=. if maxmvyr>=2005 & maxmvyr!=.

bysort pidp: gen bhpsmiss=1 if maxmvyr<0 & bhps==2

bysort pidp: gen ukhlsmiss=1 if maxmvyr<0 & bhps==1

*Creating an indicator to restrict to those who have minimum wave<12 for whom we assume they have been in the same LSOA since their first wave (extreme case from wave 1 to 12, although we cannot account for this)

bysort pidp: replace bhpsmiss=. if minwave<=12

bysort pidp: replace ukhlsmiss=1 if maxmvyr<0 & bhps==0

bysort pidp: replace memelsoa=0 if bhpsmiss==1 | ukhlsmiss==1

*restricting to those who have minimum wave=12 for whom we are sure they have been in the same since 2006

bysort pidp: egen minwave_new=min(wave)

bysort pidp: gen bhpsmiss_rest=1 if maxmvyr<0 & bhps==2

bysort pidp: replace bhpsmiss_rest=. if minwave_new<=15

gen memelsoa_rest=memelsoa

bysort pidp: replace memelsoa_rest=0 if bhpsmiss_rest==1 | ukhlsmiss==1

**race

gen british=1 if maxrace==1

replace british=0 if maxrace>1 & maxrace!=.

replace british=5 if british==.

replace british=1 if maxrace_bh==1 & british==.

replace british=1 if maxrace_bh==1 & british==5

replace british=0 if maxrace_bh>1 & maxrace_bh!=. & british==5

*using the cross-sectional weight for wave 26

gen weight= indinui_xw if wave==26

//Merging with demographic info about the LSOA, obtained from the 2001 and 2011 census

merge m:1 lsoa01 using demographic

drop if _merge==2

//personality traits

bysort pidp: egen maxopen21=max( big5o_dv)

bysort pidp: egen maxagr21=max( big5a_dv )

bysort pidp: egen maxconscious21=max( big5c_dv )

bysort pidp: egen maxnerv21=max( big5n_dv )

bysort pidp: egen maxextr21=max( big5e_dv )

//Dependent variables

//Brexit

gen leaveeur=0 if eumem==1

replace leaveeur=1 if eumem==2

//New independent continuous variables

gen nonbrit01=1- WhiteBritishpct_LSOA_01

gen nonbrit11=1- WhiteBritishpct_LSOA_11

gen diffnonbrit=nonbrit11-nonbrit01

egen diffnonbrit_new=std(diffnonbrit) if british==1 & memelsoa_rest==1 & wave==26 & leaveeur!=. & maxopen21!=. & weight!=0 & weight!=.

egen nonbrit11_new=std(nonbrit11) if british==1 & memelsoa_rest==1 & wave==26 & leaveeur!=. & weight!=0 & weight!=.

**Controls

**age

gen age=age_dv

//there are 37 who have age==-9, what to do with them?//

**gender

replace sex=sex_dv if sex<0

gen female=1 if sex==2

replace female=0 if sex==1

recode female (.=5)

//education//

*generating dummy for education

recode hiqual_dv (-7=.) if wave==12

gen educ=2 if hiqual_dv==1 | hiqual_dv==2

replace educ=1 if hiqual_dv==3 | hiqual_dv==4

replace educ=0 if hiqual_dv==9

replace educ=3 if hiqual_dv==5

replace educ=5 if hiqual_dv==-8 | hiqual_dv==-9 | hiqual_dv==.

recode educ (.=5)

//it-s missing the -7 in the wave 12. need to include but I dont know the label now//

label def educ4 0"Low Educ" 1"Medium Educ" 2 "High Educ" 3"Other" 5"Missing"

label values educ educ4

**Urban

gen urban=1 if urban_dv==1

replace urban=0 if urban_dv==2

recode urban (.=5)

*Tenure*

*0 if owned (with and without mortgage); 1 any paid rent; 2 social housing; 3 other or missing

gen tenure=0 if tenure_dv==1 | tenure_dv==2

replace tenure=1 if tenure_dv==5 | tenure_dv==6 | tenure_dv==7

replace tenure=2 if tenure_dv==3 | tenure_dv==4

replace tenure=3 if tenure_dv==8

replace tenure=5 if tenure_dv==. | tenure_dv==-9

label def teno 0"Owned" 1"Rented" 2"Social tenants" 3"Other" 5"Missing"

label values tenure teno

tab tenure, m

recode tenure (2=1) (1=2)

label def tennew 0"Own home" 1"Social Renting" 2"Private Renting" 3"Other" 5"Missing"

label values tenure tennew

// marital status//

gen marstatnew=0 if marstat_dv==6

replace marstatnew=1 if marstat_dv==1 | marstat_dv==2

replace marstatnew=2 if marstat_dv==5

replace marstatnew=3 if marstat_dv==4

replace marstatnew=4 if marstat_dv==3

recode marstatnew (.=11) if wave==26

label def partlabel 0"Single/Never Married" 1"Married/Living as a couple" 2"Separated" 3"Divorced" 4"Widowed" 11"Partner: Missing"

label values marstatnew partlabel

ta marstatnew, gen(marstat_)

*regional variable

gen regarea_n=gor_dv

//children in hh//

gen haschild=0 if nchild_dv==0

replace haschild=1 if nchild_dv!=0 & nchild_dv!=.

recode haschild (.=5) if wave==26

label def childr 0"Child in HH: No" 1"Child in HH: Yes" 5"CHild in HH: Missing"

label values haschild childr

//employment - to check// 1 is employed, 0 is unemployed.

gen employed=1 if jbhas==1

replace employed=0 if jbhas==2

replace employed=1 if (employed==0 | employed==.) & jbsemp==1 | jbsemp==2

replace employed=1 if (employed==0 | employed==.) & jboff==1

recode employed (.=5)

label def emplab 0"Unemployed" 1"Employed" 5"Employment: Missing"

label values employed emplab

//NS_sec

replace jbnssec5_dv=1 if wave<19 & jbnssec_dv>0 & jbnssec_dv<13

replace jbnssec5_dv=2 if wave<19 & jbnssec_dv>12 & jbnssec_dv<17

replace jbnssec5_dv=3 if wave<19 & jbnssec_dv>16 & jbnssec_dv<21

replace jbnssec5_dv=4 if wave<19 & jbnssec_dv>20 & jbnssec_dv<24

replace jbnssec5_dv=5 if wave<19 & jbnssec_dv>23 & jbnssec_dv<=35

gen sec=jbnssec5_dv

recode sec (.=13) (-8=13) (-9=13)

recode sec

label def sec1 1"Management & professional" 2"Intermediate" 3"Small employers & own account" 4" Lower supervisory" 5"Semi-routine & routine" 13"Missing"

label values sec sec1

** Two compositional variables of the neighbourhood

gen neighcomposition_old=Age65pluspct_LSOA_11

//Deprivation index

factor EcActUnemppct_LSOA_11 HHsocrentpct_LSOA_11 FemaleLPHHpct_LSOA_11, factor(1)

predict deprivation

xtile depriv_dec=deprivation, nq(10)

//generating interactions

gen diffagr=maxagr21*diffnonbrit_new

gen diffagrl=maxagr21*nonbrit11_new

gen diffopen=maxopen21*diffnonbrit_new

gen diffopenl=maxopen21*nonbrit11_new

gen diffnerv=maxnerv21*diffnonbrit_new

gen diffnervl=maxnerv21* nonbrit11_new

gen diffconsc=maxconscious21*diffnonbrit_new

gen diffconscl=maxconscious21* nonbrit11_new

gen diffextr=maxextr21*diffnonbrit_new

gen diffextrl=maxextr21* nonbrit11_new

tab regarea, gen (regarea_)

tab employed, gen (employed_)

tab educ, gen (education_)

tab tenure, gen (tenure_)

ta marstatnew, gen (marstatnew_)

ta haschild, gen (haschild_)

ta sec, gen (sec_)

ta urban, gen (urban_)

//Descriptives statistics

mac def variables "leaveeur diffnonbrit nonbrit11 deprivation neighcomposition_old maxagr21 maxextr21 maxconscious21 maxnerv21 maxopen21 age female haschild_* tenure_* marstatnew_* employed_* sec_* education_* urban_* regarea_* "

estpost tabstat $variables if british==1 & wave==26, stats(Mean SD) columns(statistics)

est sto a_2

estpost tabstat $variables if memelsoa_rest==1 & british==1 & wave==26, stats(Mean SD) columns(statistics)

est sto b_2

qui logit leaveeur c.diffnonbrit_new c.nonbrit11_new c.maxagr21 maxextr21 maxconscious21 maxnerv21 maxopen21 age female ib0.haschild ib0.tenure ib0.marstatnew ib1.employed ib5.sec ib0.educ ib0.urban ib1.regarea deprivation neighcomposition_old if memelsoa_rest==1 & british==1 [pweight= weight], cluster(LSOA) or

estpost tabstat $variables if e(sample), stats(Mean SD) columns(statistics)

est sto c_2

esttab *_2 using descriptives_nw.rtf, main(Mean) nogap one aux(SD) b(3) modelwidth(4) mlabels("Full sample" "Restricted" "Empirical" ) title("Table 1: Descriptive Statistics, mean(sd)") label replace

//Models for table 2

qui logit leaveeur c.diffnonbrit_new c.nonbrit11_new c.maxagr21 maxextr21 maxconscious21 maxnerv21 maxopen21 age female ib0.haschild ib0.tenure ib0.marstatnew ib1.employed ib5.sec ib0.educ ib0.urban ib1.regarea deprivation neighcomposition_old if memelsoa_rest==1 & british==1 [pweight= weight], cluster(LSOA) or

outreg2 using baseline_sco.rtf, replace ctitle( Brexit, nointeractions) label dec(3) pdec(3) alpha(0.001, 0.01, 0.05, 0.1) symbol(***, **, *, +) eform e(r2_p)

qui logit leaveeur c.diffnonbrit_new##c.maxagr21 c.diffnonbrit_new##c.maxextr21 c.diffnonbrit_new##c.maxconscious21 c.diffnonbrit_new##c.maxnerv21 c.diffnonbrit_new##c.maxopen21 age female ib0.haschild ib0.tenure ib0.marstatnew ib1.employed ib5.sec ib0.educ ib0.urban ib1.regarea deprivation neighcomposition_old nonbrit11_new if memelsoa_rest==1 & british==1 [pweight=weight], cluster(LSOA) or

outreg2 using baseline_sco.rtf, ctitle( Brexit, all inter_change) label dec(3) pdec(3) alpha(0.001, 0.01, 0.05, 0.1) symbol(***, **, *, +) eform e(r2_p)

qui logit leaveeur c.nonbrit11_new##c.maxagr21 c.nonbrit11_new##c.maxextr21 c.nonbrit11_new##c.maxconscious21 c.nonbrit11_new##c.maxnerv21 c.nonbrit11_new##c.maxopen21 age female ib0.haschild ib0.tenure ib0.marstatnew ib1.employed ib5.sec ib0.educ ib0.urban ib1.regarea deprivation neighcomposition_old c.diffnonbrit_new if memelsoa_rest==1 & british==1 [pweight=weight], cluster(LSOA) or

outreg2 using baseline_sco.rtf, ctitle( Brexit, all inter_levels) label dec(3) pdec(3) alpha(0.001, 0.01, 0.05, 0.1) symbol(***, **, *, +) eform e(r2_p)

//Graphs

egen p05 = pctile(diffnonbrit_new), p(05)

egen p95 = pctile(diffnonbrit_new), p(95)

egen p05_l = pctile(nonbrit11_new), p(05)

egen p95_l = pctile(nonbrit11_new), p(95)

*figure 1a

qui logit leaveeur c.nonbrit11_new##c.maxagr21 c.nonbrit11_new##c.maxextr21 c.nonbrit11_new##c.maxconscious21 c.nonbrit11_new##c.maxnerv21 c.nonbrit11_new##c.maxopen21 age female ib0.haschild ib0.tenure ib0.marstatnew ib1.employed ib5.sec ib0.educ ib0.urban ib1.regarea deprivation neighcomposition_old c.diffnonbrit_new if memelsoa_rest==1 & british==1 [pweight=weight], cluster(LSOA) or

qui margins, at (nonbrit11_new= (-.648794 2.090217) maxagr21 = (1 7))

marginsplot

save agr_lev

*figure 1b

qui logit leaveeur c.diffnonbrit_new##c.maxagr21 c.diffnonbrit_new##c.maxextr21 c.diffnonbrit_new##c.maxconscious21 c.diffnonbrit_new##c.maxnerv21 c.diffnonbrit_new##c.maxopen21 age female ib0.haschild ib0.tenure ib0.marstatnew ib1.employed ib5.sec ib0.educ ib0.urban ib1.regarea deprivation neighcomposition_old nonbrit11_new if memelsoa_rest==1 & british==1 [pweight=weight], cluster(LSOA) or

qui margins, at (diffnonbrit_new= (-.8168518 2.069168) maxagr21 = (1 7))

marginsplot

save agr_diff

*figure 2a

qui logit leaveeur c.nonbrit11_new##c.maxagr21 c.nonbrit11_new##c.maxextr21 c.nonbrit11_new##c.maxconscious21 c.nonbrit11_new##c.maxnerv21 c.nonbrit11_new##c.maxopen21 age female ib0.haschild ib0.tenure ib0.marstatnew ib1.employed ib5.sec ib0.educ ib0.urban ib1.regarea deprivation neighcomposition_old c.diffnonbrit_new if memelsoa_rest==1 & british==1 [pweight=weight], cluster(LSOA) or

qui margins, at (nonbrit11_new= (-.648794 2.090217) maxopen21 = (1 7))

marginsplot

save open_lev

*figure 2b

qui logit leaveeur c.diffnonbrit_new##c.maxagr21 c.diffnonbrit_new##c.maxextr21 c.diffnonbrit_new##c.maxconscious21 c.diffnonbrit_new##c.maxnerv21 c.diffnonbrit_new##c.maxopen21 age female ib0.haschild ib0.tenure ib0.marstatnew ib1.employed ib5.sec ib0.educ ib0.urban ib1.regarea deprivation neighcomposition_old c.diffnonbrit_new if memelsoa_rest==1 & british==1 [pweight=weight], cluster(LSOA) or

qui margins, at (diffnonbrit_new= (-.8168518 2.069168) maxopen21 = (1 7))

marginsplot

save open_diff

*figure 1

grc1leg2 agr_lev agr_diff

*figure 2

grc1leg2 open_lev open_diff

1. We excluded those who did not vote from the analysis but also tested coding them as leave supporters or remain supporters, but substantively similar findings were returned. [↑](#footnote-ref-1)
2. In other words, the continuous variable does not run from 0 to 100 but from 0 to 1, e.g., 0=0%, 0.25=25%, 0.6=60%, etc. [↑](#footnote-ref-2)
